# Underwater In Situ Dissolved Gas Detection Based on Multi-Reflection Raman Spectroscopy

**DOI:** 10.3390/s21144831

**Published:** 2021-07-15

**Authors:** Meng Li, Qingsheng Liu, Dewang Yang, Jinjia Guo, Ganshang Si, Lulu Wu, Ronger Zheng

**Affiliations:** 1 College of Information Science and Engineering, Ocean University of China, Qingdao 266100, China; limeng@stu.ouc.edu.cn (M.L.); liuqingsheng@stu.ouc.edu.cn (Q.L.); oucsgs0903@126.com (G.S.); wululu@stu.ouc.edu.cn (L.W.); rzheng@ouc.edu.cn (R.Z.); 2 College of Ocean Science and Engineering, Shandong University of Science and Technology, Qingdao 266100, China; yangdewang_lcu@126.com

**Keywords:** Raman spectroscopy, underwater in situ detection, dissolved gas, multi-reflection

## Abstract

The detection of dissolved gases in seawater plays an important role in oceanic observations and exploration. As a potential technique for oceanic applications, Raman spectroscopy has been successfully applied in hydrothermal vents and cold seep fluids, but it has not yet been used in common seawater due to the technique’s lower sensitivity. In this work, we present a highly sensitive underwater in situ Raman spectroscopy system for dissolved gas detection in common seawater. Considering the difficulty of underwater degassing and in situ detection, we designed a near-concentric cavity to improve the sensitivity, with a miniature gas sample chamber featuring an inner volume of 1 mL placed inside the cavity to reach equilibrium in a short period of time. According to the 3σ criteria, the detection limits of this system for CO_2_, O_2_, and H_2_ were calculated to be 72.8, 44.0, and 27.7 ppm, respectively. Using a hollow fiber membrane degasser with a large surface area, the CO_2_ signal was found to be clearly visible in 30 s at a flow rate of 550 mL/min. Moreover, we deployed the system in Qingdao’s offshore seawater, and the field test showed that this system is capable of successfully detecting in situ the multiple gases dissolved in the seawater simultaneously.

## 1. Introduction

The detection of dissolved gases in seawater plays an important role in oceanic observations and exploration and is essential for studying the ocean’s environment and ecosystem [1]. For example, CO_2_ [2] is a key factor in global warming, and O_2_ [3] is an important sign of net biological oxygen production. There is a certain relationship between CO_2_ and O_2_ in primary production (photosynthesis and chemosynthesis) and secondary production (respiration) [4]. Dissolved H_2_ is a key parameter of thermodynamic equilibria and kinetic processes in water–rock interactions process [5]. Thus, there is significant scientific and environmental value in tracking the concentrations of dissolved gases in the ocean. However, the low concentrations of dissolved gases and the complex oceanic environment are significant challenges for in situ dissolved gases sensors.

Recently, many in situ dissolved gas sensors have been reported, such as dissolved oxygen (DO) sensors based on the luminescent or electrochemical methods [6,7,8,9], CO_2_ sensors based on infrared absorption spectroscopy [10,11,12,13,14], and H_2_ sensors based on the electrochemical method [15,16,17]. While most of these sensors can only measure a single gas, underwater mass spectrometers and Raman spectrometer can achieve in situ measurements for the simultaneous measurement of multiple gases. Mass spectrometers require a high vacuum, which makes it difficult for such devices to work for a long period of time in water. Further, mass spectrometers are not able to distinguish between some molecules with the same mass [18,19]. Raman spectroscopy can detect multiple gases simultaneously. This method has been successfully applied in hydrothermal vents and cold seep fluids for dissolved gas detection and obtained many results that were different from the sampling measurements [20,21,22,23]. However, this method can be used only in high concentration areas due to its lower sensitivity, thus, the background concentrations of ocean water have not yet been measured. It would be ideal if the marine applications of Raman spectroscopy could realize the measurement of multiple gases dissolved in common seawater, after improving and integrating the technology with existing deep sea Raman spectroscopy systems.

There are many methods for enhancing Raman sensitivity, such as surface-enhanced Raman spectroscopy (SERS) [24] and resonance Raman scattering [25]. These two methods significantly enhance the Raman signal based on enlarging the scattering cross section. However, neither of them is easily applicable to in situ dissolved gases, due to sample pretreatment. The multi-pass cavity method is comparatively more effective and practical in improving the sensitivity and has broad potential in dissolved gas detection. This method has been widely used in absorption spectroscopy [26,27] and was first applied to Raman spectroscopy in 1974 [28]. Since then, numerous types of cavities have been applied to enhance the Raman signals of various gases. Li et al. developed a near-confocal cavity owing to the beam being reflected 50 times, and high-sensitivity detection of a mixture of eight gases (i.e., H_2_, N_2_, CH_4_, C_2_H_2_, C_2_H_4_, C_2_H_6_, CO_2_, and CO) with the same volume ratio has been achieved [29,30]. Taylor et al. built an actively stabilized external resonator using a concentric cavity and achieved a 50-fold enhancement of gaseous Raman signals, and the resulting limit of detection (LOD) for H_2_ in ambient-pressure gas mixtures is about 10 ppm in a 1 min analysis period at a unity signal-to-noise ratio [31]. Utsav et al. used a concentric cavity and achieved more than a 16-fold enhancement in Raman signals for a flame [32]. Wang et al. invented a V-shaped cavity to enhance the Raman signal intensity; with a 20 s exposure time, ppm-level gas sensing was achieved under 1 bar of total pressure, including CO_2_, CO, H_2_, CH_4_, C_2_H_6_, C_2_H_4_, C_2_H_2_, N_2,_ and O_2_ [33]. Liu et al. developed a folded near-concentric cavity with 68 reflections and achieved about a 1.5-times enhancement compared with an unfolded near-concentric cavity [34]. Yang et al. developed a multi-reflection, cavity-enhanced Raman spectroscopy (CERS) probe for in situ multi-component gas detection, with LODs of CH_4_, H_2_, CO_2,_ and water vapor of 44.5 ppm, 192.9 ppm, 317.5 ppm, and 0.67%, respectively [35].

Inspired by the good results of these studies, we also designed a near-concentric cavity and achieved LODs of 2.32 and 0.44 μmol/L for CO_2_ and CH_4_, respectively, in the lab. A sample chamber made of aluminum, with dimensions of 40 × 30 × 40 mm and an inner volume of 10 mL was used to collect the gas samples. A test experiment was also carried out with a gas–liquid separator coupled to the Raman system, and signals of O_2_ and CO_2_ were detected after 1 h of degasification [36]. This system has shown potential for gas detection in water. In this paper, the previous system was miniaturized, and all components were integrated into the pressure vessel to achieve underwater in situ measurements. Through optimization of the near-concentric cavity and gas sample chamber, the volume of gas to be measured was reduced, and the detection efficiency was improved. Tests were primarily carried out in the laboratory and in nearshore water. The LODs of the system for O_2_, CO_2,_ and H_2_ and the degassing efficiency at different flow rates were measured. The Raman signals of O_2_, CO_2_, H_2_, and N_2_ dissolved in seawater were also measured in the nearshore test.

## 2. System Configurations

**Figure 1:**
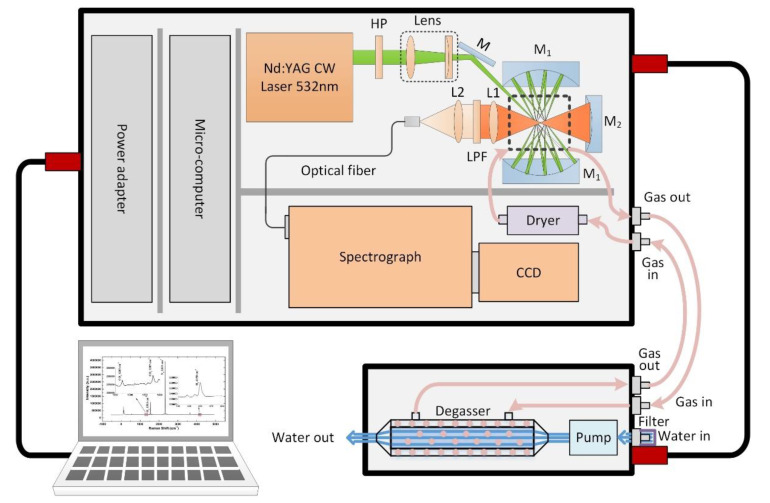
Framework of the in situ underwater Raman system for dissolved gas detection.

The configuration of the in situ underwater Raman system for dissolved gas detection is shown in Figure 1. This system consists of a degassing vessel and a detecting vessel. The components in the degassing vessel and the detecting vessel are packaged in pressure vessels (380 mm long by 133 mm in diameter and 790 mm long by 256 mm in diameter) capable of withstanding 2 MPa within a 50% safety margin. The vessels were machined from 6061 aluminum alloy and hard anodized to retard corrosion. The total weight of these two assembled pressure vessels is 60 kg in air and 18 kg in water. Two waterproof connectors (6-pin and 2-pin) and two gas-type fittings are installed in the front-end cap of the detecting vessel. A 2-pin waterproof connector, a water pipe connector, and two gas-type fittings are installed in the front-end cap of the degassing vessel, and a water pipe connector is installed in the rear-end cap of the degassing vessel. Through the 6-pin connector and a pressure-tolerant cable, the Raman system connects to a shore-based system for power supply, system control, and signal delivery. Through the two 2-pin connectors and a cable, the degassing vessel connects to the detecting vessel to power the degassing vessel. Two water pipe connectors are used for the water inlet and outlet, and there is a filter installed in the inlet to keep impurities out.

Inside the degassing vessel, there is a water pump and a degasser. The water is pumped into the pipeline within the degassing vessel through the pump, passing through the filter and flowing into the degasser. The filter, a stainless-steel mesh with a precision of 10 μm, is used to filter the biological debris, sediments and other large particles in seawater to prevent them from entering into the pump and degasser, and thus, affecting the service life and degassing efficiency of multiple components in the system. The degasser (3M™ Liqui-Cel™, 1.7 × 5.5 MiniModule) is a membrane contactor that uses microporous hollow fiber membranes that are knitted into an array and wrapped around a center tube inside the contactor housing. The hollow fiber membrane is made of polypropylene, with a surface area of 0.54 m^2^ and a maximum liquid flow rate of 2.5 L/min. During operation, liquid flows around one side of the membrane and then gas is obtained on the other side of the membrane. Since the microporous membrane is hydrophobic, the membrane will not allow liquid water to pass through the pores into the gas side of the membrane [37]. The large surface area and high liquid flow rate provide a faster degassing rate and make it possible for rapid measurements in seconds. By using four gas-type fittings and two capillary tubes made of stainless steel, the degassing vessel and the detecting vessel circulate gas to shorten the gas–liquid equilibrium time.

**Figure 2:**
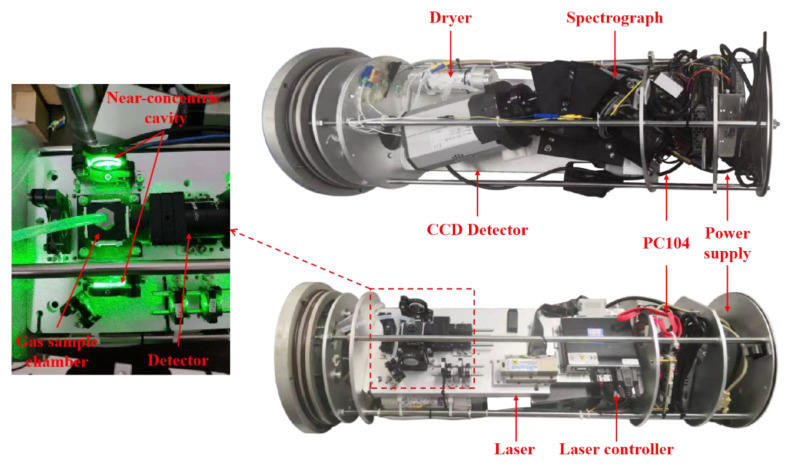
Top and bottom views of the detecting vessel.

Inside the detecting vessel, there is a dryer, a gas sample chamber, a continuous-wave (CW) laser, an optics module, a high-throughput spectrograph, a charge-coupled device (CCD) detector, a PC-104-embedded computer, and an electronics module. These units are mounted on both sides of an optical bench, which are fixed in place by the internal restriction slot during operation. The CW laser used as the Raman excitation source is a diode-pumped, frequency-doubled Nd:YAG CW laser (532 nm) with power of 300 mW. The polarization of the laser beam is rotated by 90° by passing it through a half-wave plate (HP), and then the diameter of the laser beam can be compressed and focused into the chamber by a couple of lenses (a planoconvex lens with a focal length of 100 mm and a planoconcave lens with a focal length of −75 mm). The near-concentric cavity is composed of two identical spherical mirrors that are 25.4 mm in diameter with a 25 mm focal length, and was achieved by rotating a second mirror clockwise by about 0.05° and 40-times reflections. A gas sample chamber with optical windows is mounted at the center of the cavity. In the analysis, scattering signals from the center of the cavity were collected by an achromatic doublet lens L1 (*f* = 30 mm) with a diameter of 25.4 mm. A mirror (*f* = 12.5 mm) placed on the opposite side from the sample chamber was used to enhance the collection efficiency of the signal. Another identical lens L2 (*f* = 30 mm) was then used to couple the Raman signal into the delivery optical fiber bundle (19 × 100 μm, NA = 0.22) with a long-pass filter (LPF), whose cutoff wavelength is 540 nm, placed in front to remove Rayleigh scattering from the collected signal. From the other end of the fiber, the signal was then coupled into a spectrograph. The spectrograph (P&P Optica, 532 nm Raman, Waterloo, Canada) used for Raman scattering light splitting is specialized for underwater Raman measurements with a 3 f/#, 20 μm width entrance slit and 1300 lines/mm grating. The Raman spectra can then be recorded by a CCD (Andor, iDus 416, Abingdon, UK) detector with a 2000 × 256 imaging array and a 15 × 15 μm pixel size operating at −70 °C. The spectral range is −600–4900 cm^−1^ with a resolution of 8 cm^−1^. Considering the difficulty of underwater degassing and in situ detection, the gas sample chamber made of stainless steel has an inner volume of just 1 mL, with dimensions of 15 × 16 × 20 mm, to shorten the gas–liquid equilibrium time. Four separate optical windows are mounted on the other four sides of the chamber for optical alignment. To minimize the influence on the scattered signal, the windows on each side of the chamber are coated with an anti-reflection coating (the transmission is 99% at 532 nm), with dimensions of 5 mm in diameter and 2 mm in thickness. Two gas tubes, the inlet and the outlet, are mounted at the top and bottom of the chamber. The gas dissolved in the water is collected by the degasser in the degassing vessel, dehumidified by the dryer, and then pumped into the chamber to be detected. The dryer is a transparent acrylic tube filled with anhydrous calcium chloride and cobalt chloride. Anhydrous calcium chloride is used for dehumidification, and cobalt chloride is used as an indicator. When the color of cobalt chloride changes, the drying effect is no longer achieved, and a new desiccant needs to be replaced. The detailed specifications of the Raman system are shown in Table 1. A PC104 microcomputer was used to control the laser and the CCD detector. This microcomputer also communicates with the computer on deck via Ethernet. The operational parameters of the laser and the CCD detector can be set up using the computer on deck. An internal view of the Raman system is shown in Figure 2.

## 3. Results and Discussion

To evaluate the performance of the in situ underwater Raman system for dissolved gas detection, systematic experiments were conducted in our lab and at the wharf.

**Figure 3:**
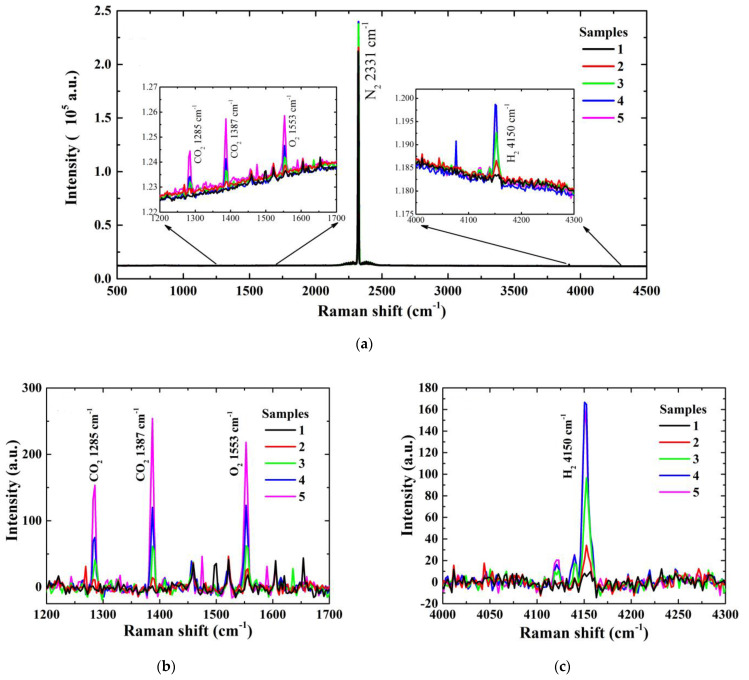
Raman original spectra of standard gas with 5 typical concentrations (**a**), and the spectra after baseline subtraction of CO_2_ and O_2_ (**b**) and H_2_ (**c**).

**Figure 4:**
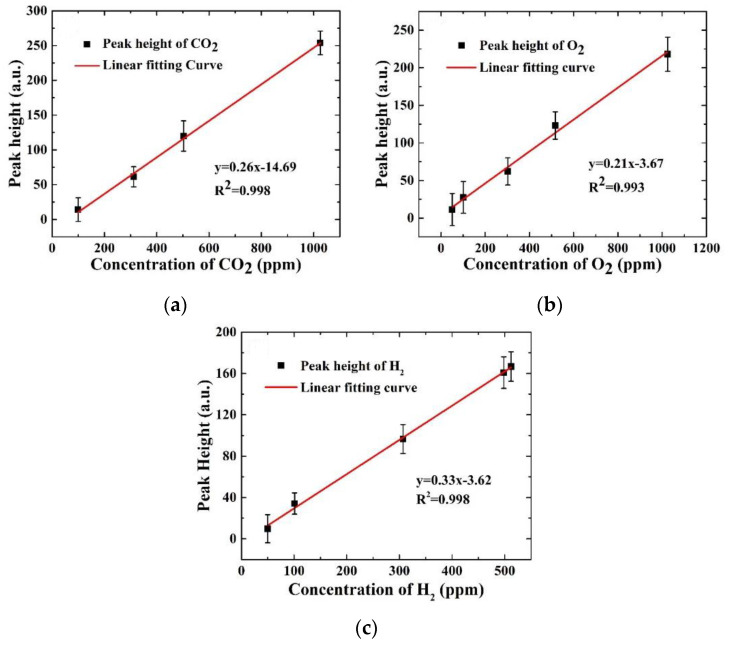
Linear relationships between concentrations and peak intensity of CO_2_ (**a**), O_2_ (**b**), and H_2_ (**c**).

First, to evaluate the performance of the detecting vessel, especially the near-concentric cavity, we collected the Raman signals of five different concentrations of gas samples, as shown in Table 2. To obtain a relatively desirable Raman signal and prevent overexposing the CCD, each spectrum was accumulated 10 times with an acquisition time of 10 s, and 20 measurements were taken for each sample to evaluate repeatability. Five original spectra of typical concentrations are shown in Figure 3a, and the spectra after baseline subtraction of CO_2_, O_2_ and H_2_ are shown in Figure 3b,c. In Figure 3, the 100 ppm CO_2_, 50 ppm O_2,_ and 50 ppm H_2_ Raman signals can be clearly observed. The linear relationship between concentrations and peak intensity of the 1387 cm^−1^ CO_2_, 1553 cm^−1^ O_2,_ and 4150 cm^−1^ H_2_ signal are shown in Figure 4. Since the signal of 50 ppm CO_2_ is weak, either 1285 cm^−1^ or 1387 cm^−1^, the linear relationship of CO_2_ in Figure 4a is only fitted with the peak heights of the other four concentrations. The responses of the Raman system for CO_2_, O_2,_ and H_2_ detection are linearly good, with R^2^ = 0.998, 0.993, and 0.997 over the whole range, as shown in Figure 4. The LODs of CO_2_, O_2,_ and H_2_ with three times signal-to-noise ratio (SNR) were calculated as 72.8, 44.0, and 27.7 ppm, respectively.

**Figure 5:**
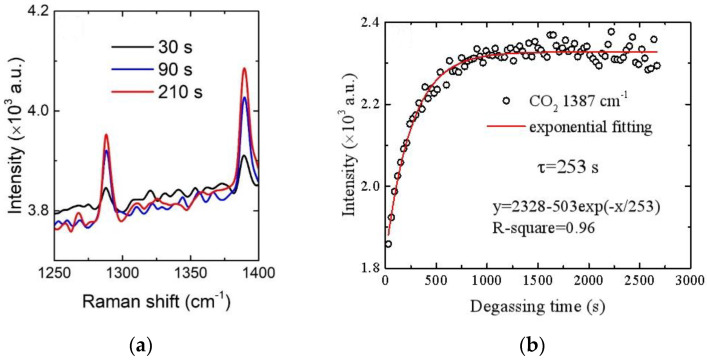
Spectra of different degassing times (**a**) and response curves of CO_2_ signal intensity with degassing time (**b**) at 550 mL/min.

Secondly, to evaluate the performance of the degassing vessel and the effect of the miniature gas sample chamber, we collected the Raman spectroscopy signals in the gas chamber degassed with tap water as time progressed, especially the CO_2_ signal whose concentration was relatively low. During the experiment, two valves were connected to the inlet and outlet of the gas chamber, respectively, and a vacuum pump was connected behind the outlet valve. First, the inlet valve was closed and the outlet valve and vacuum pump were opened, until the gas chamber was pumped to near vacuum, and then the outlet valve was closed and the inlet valve and water pump were opened, so that the gas degassed from tap water by the degasser entered the gas chamber and accumulated continually. The spectra of three different degassing times (30, 90, and 210 s) at 550 mL/min are shown in Figure 5a, and the response curve of the CO_2_ signal intensity with degassing time at 550 mL/min is shown in Figure 5b. In Figure 5, the CO_2_ signal is clearly visible at 30 s. With an increase in time, the CO_2_ signal strength gradually increases exponentially. This is because as gas molecules move across the membrane and into the gas chamber, the concentration gradient decreases, and as a result, the rate of gas flux across the membranes slows. The instantaneous rate of equilibration is directly proportional to the magnitude of the gradient and this means that the equilibration will be described by an exponential function. We also can see from Figure 5b that the time constant for equilibration (i.e., the response time) was determined to be τ = 253 s based on exponential fitting.

**Figure 6:**
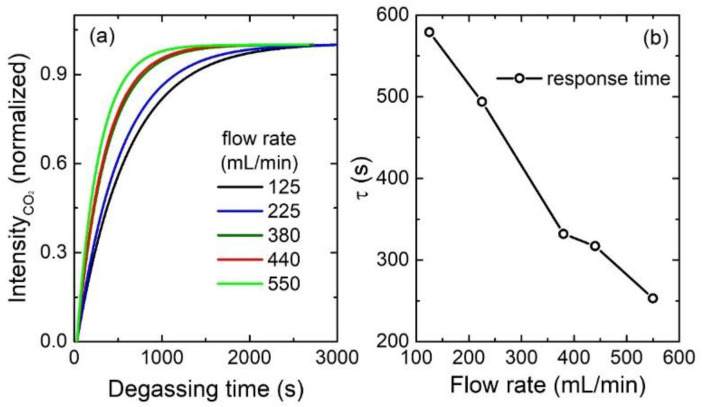
The fitted curves of CO_2_ signal intensity with degassing time at different flow rates (**a**); the response time variation with the flow rate (**b**).

We also collected the CO_2_ signals in the gas chamber degassed with tap-water at different flow rates as time progressed. The fitted curves of CO_2_ signal intensity with the degassing time at five different flow rates (125, 225, 380, 440, and 550 mL/min) are shown in Figure 6a. The response time’s variation with the flow rate is shown in Figure 6b and Table 3. As shown in Figure 6 and Table 3, as the flow rate increases, it takes less time for the CO_2_ to reach equilibrium. Therefore, the equilibrium time can be further reduced by increasing the flow rate.

**Figure 7:**
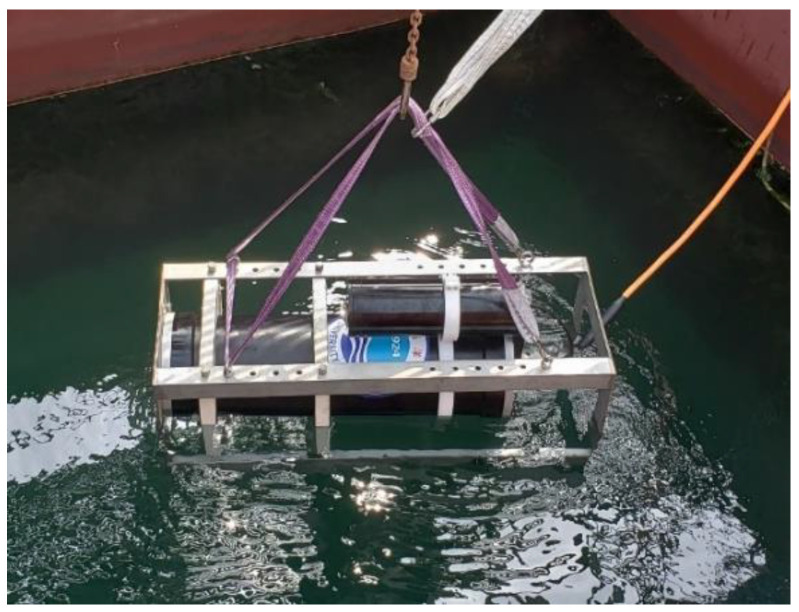
Photo of the field experiment.

**Figure 8:**
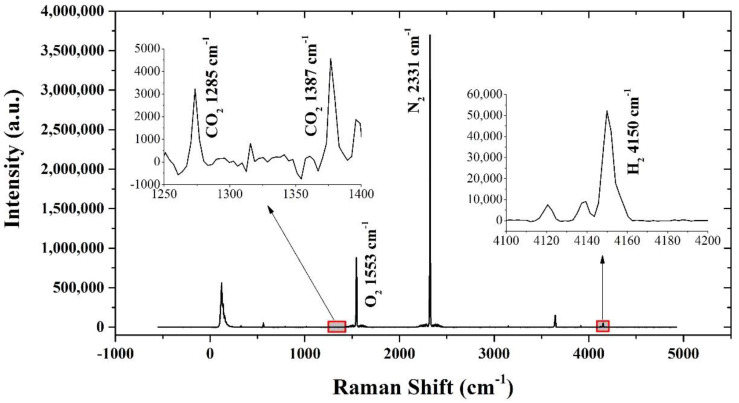
A typical Raman spectrum of dissolved gases in Qingdao’s offshore seawater.

After evaluating the performance of the Raman system in the lab, we deployed the system in seawater at the Qingdao Zhongyuan Wharf in June 2019; the system was powered by a cable from the shore. A photo of the field experiment is shown in Figure 7. A typical Raman spectrum of dissolved gases in seawater after baseline subtraction is shown in Figure 8. It is important to note that the change in dissolved gas concentration in the seawater is relatively slow, and the field experiment did not require a high sampling frequency. Therefore, the parameters were adjusted appropriately according to the spectrum obtained in the field. The acquisition time was set to the maximum under the premise of ensuring unsaturation (11 s), and the cumulative time was increased to 60 times. Figure 8 shows the significant signals of N_2_ and O_2_, and the signals of CO_2_ and H_2_ are also clearly visible after partial magnification. Thus, it was shown that the system is capable of simultaneously detecting multiple gases (CO_2_, O_2_, N_2,_ and H_2_) in seawater. In the near future, the system will be further optimized in terms of its hardware and data processing, and more scientific application studies will be carried out.

## 4. Conclusions

To simultaneously measure the multiple gases dissolved in common seawater, we developed a highly sensitive underwater in situ Raman spectroscopy system based on multiple reflections. This system consists of a degassing part and a detecting part, housed in a L380 × Φ133 mm pressure vessel and a L790 × Φ256 mm pressure vessel, respectively, with a total weight of 60 kg. Based on the special designs of the near-concentric cavity and the miniature gas sample chamber placed in the cavity with an inner volume of 1 mL, the LODs of CO_2_, O_2,_ and H_2_ with three-times SNR were determined to be 72.8, 44.0, and 27.7 ppm, respectively. Combined with a hollow fiber membrane degasser featuring a large surface area, the CO_2_ signal with a relatively low concentration was clearly visible at 30 s, and the time constant for equilibration was 253 s at a flow rate of 550 mL/min. As the flow rate increased, the dissolved gases required less time to reach equilibrium. After evaluating the performance of the Raman system in a lab, we deployed the system in Qingdao’s offshore seawater and acquired Raman signals for N_2_, O_2_, CO_2,_ and H_2_ in seawater. The field test preliminarily demonstrated the capability of the system to simultaneously detect multiple gases in situ. In the near future, the system will be further optimized, and more scientific application studies will be carried out.

## Figures and Tables

**Table 1:** Specifications of the in situ underwater Raman system for dissolved gas detection.

Module	Apparatus	Specifications
Mechanics	vessel	6061 aluminum alloy, anodized surface L 380 × D 133 mm (degassing vessel) L 790 × D 256 mm (detecting vessel) 60 kg/weight in air 2 MPa/pressure-proof
Gas–liquid separation	degasser	0.54 m^2^/membrane surface area 2.5 L/min/maximum liquid flow rate
	dryer	anhydrous calcium chloride and cobalt chloride
	gas sample chamber	stainless steel 15 × 16 × 20 mm/dimensions 1 mL/inner volume
Optics	laser	532 nm, Nd:YAG 300 mW/power
	near-concentric cavity	25.4 mm/diameter of the spherical mirrors 25 mm/focal length of the spherical mirrors
	detector	2000 × 256/active pixels 15 × 15 μm/pixel size −70 °C/cooling temperature
	spectrograph	−600–4900 cm^−1^/spectral range 8 cm^−1^/spectral resolution
Electronics	power supply	220VAC from the shored-based system
	microcomputer	PC 104/Advantech
	communication	Ethernet Remote desktop connection

**Table 2:** Composition and content of gas samples.

Sample	C(CO_2_)/ppm	C(O_2_)/ppm	C(H_2_)/ppm	C(N_2_)
1	50.5	50.8	50	other
2	99.6	101	101	other
3	312	302	307	other
4	503	517	512	other
5	1025.2	1025.4	498.2	other

**Table 3:** The equilibrium times at different flow rates.

Flow Rate (mL/min)	Equilibrium Time (s)
125	579
225	494
380	332
440	317
550	253

## Data Availability

Data sharing not applicable.
